# Editorial: The Sociocultural Context of Psychosocial Interventions

**DOI:** 10.3389/fpsyg.2018.01795

**Published:** 2018-09-24

**Authors:** Francisco José Eiroa-Orosa

**Affiliations:** ^1^Section of Personality, Evaluation and Psychological Treatment, Department of Clinical Psychology and Psychobiology, School of Psychology, Institut de Neurociències, Universitat de Barcelona, Catalonia, Spain; ^2^Program for Recovery and Community Health, Yale School of Medicine, Yale University, New Haven, CT, United States

**Keywords:** sociocultural context, psychosocial interventions, culture, ideology, neutrality

During the last decades several authors have criticized the arrival of evidence-based practice (EBP) approaches due to its contribution to the decontextualization of psychosocial and behavioral health interventions (Coghlan and Brydon-Miller, 2014). The term decontextualization here refers to the fact that, although it is not something explicit in its foundations (Leach, 2006), EBP researchers have in practice largely prioritized the internal over the external validity of evaluations. In other words, the problem is not the use of evidence against tradition, intuition or power; but that what has been considered “good” evidence has been narrowed to be aligned with the dogma of behavioral sciences' neutrality. In relation to this alleged neutrality, Cushman (1990) already discussed the historical configuration of the “self” concept. Psychology, he argued, has the role of “*healing the empty self (…) without being able to address its historical causes*,” (pp. 599) thus being responsible in part for the very process of “emptying” which it claims, and aims, to address. Cushman argues that psychology strives to appear as a neutral science, while its interests—its subjects, ideologies and businesses—have “historical antecedents, economic constituents, and political consequences” (p. 600). In this regard, Sampson (1989), gives evidence that the transformation of psychology as a discipline in which the functional unit of social order has moved from the community and the household to the individual level, as postmodernism and globalization have exerted their influences. The theory of the individual as a rational and self-directed entity has produced several industries that are responsible for ensuring the proper functioning of a newly disembodied self. Among these, mental health, which has focused on eliminating symptoms regardless of their source, has become one of the fastest developing industries during the past century.

It would be unfair not to recognize countless theoretical and empirical efforts carried out especially in the last 30 years by researchers who have delved into the covariates of assessment and intervention success related to class, gender, race, or culture. However, many of these aspects remain underrepresented in the training of practitioners, research budgets and outreach activities aimed at the general public. All these strategic areas are still highly biased toward individualistic (Thomas, 2016), brain-centric (Pérez Álvarez, 2011), and positivistic (Williams and Garner, 2002) views of evaluation. In spite of this, there have been well-established calls for the integration of biological, psychological and social determinants of health and wellbeing (Engel, 1977; Cloninger, 2006) and for the mixture of methods of inquiry (Poses and Isen, 1998), however the reality today is far from being balanced.

The aim of this research topic is to bring a more “contextual” mindset to the implementation and evaluation of health and wellbeing interventions. Our main objective is to contribute to the shift in the way in which such interventions are designed and implemented, both at a granular local level (i.e., influencing individual practitioners) and at a large-scale macro level (e.g., influencing policy makers).

In the selection of articles for this special topic we have considered the need to promote self-critical research on underrepresented contexts and innovations that provide greater awareness of excluded groups.

Firstly, some of the articles give special importance to the macro-sociocultural context where interventions take place. In this sense, Giordano et al. present a group analytic intervention conducted within a ‘Community Based Participatory Research’ in an area oppressed by the Mafia. Their qualitative findings revealed the development of an awareness process that allowed the participants to recognize the strong emotional impact related to the Mafia's presence in their lives. Additionally, Eiroa-Orosa and Rowe reflect on how to transfer the principles and practice of Citizenship in Mental health to different sociocultural contexts. Citizenship is a novel approach to mental health based on people's connections to the rights, responsibilities, roles, resources and relationships that society offers to them through public and social institutions. The authors evaluate the multiple possibilities that this approach has, without forgetting the neo-colonialist risks of exporting ideas from a prestigious academic institution to daily reality in a distant context. Macro factors such as gender have also been addressed in this special topic. Wang et al. performed a metanalysis on gender differences when reporting depressive symptoms in non-clinical populations. Their results appear to confirm the so-called “female preponderance” in the level of self-reported depressive symptoms in the general population, giving support to social gender role theories in explaining these differences.

On another level, other articles on this special topic address meso-levels such as schools and the peculiarities of implementing real world research in these settings. In this line, Orosz et al. present a self-critique of their Growth Mindset intervention whose positive effects were not as long lasting as they previously expected. These authors discuss how contextual factors, such as the focus on non-problematic students or the implementation of the intervention in a non-transition stage may have been the reasons for its lack of effectiveness. Similarly, Andrés-Rodríguez et al. adapted their evaluation methods in order to be conducted by teachers in the school context. This involved a curricular intervention including education and social contact with persons with lived experience of mental disorders. They discuss the advantages and limitations of implementing a flexible methodology in the classroom including social contact with stakeholders.

A topic that has attracted much attention is the immediate environment of people affected by physical and mental health problems. Considering the importance of family care, Delle Fave et al. explore happiness, goals, and meanings among persons with multiple sclerosis and their caregivers, including socio-cultural aspects that may crucially contribute to their functioning. The authors stress at positive adjustment to the disease evolves as a result of the development of personal and family resources both among persons affected as well as their caregivers. Shi et al. examine the association between family function and self-esteem of Chinese university students with and without grandparenting experience, exploring the moderating effects of social support within this relation as well. The same authors also evaluated the effect of Systemic Therapy as they compared supportive therapy to a 6-month treatment for students who were at a clinically higher risk of developing psychosis. Although no time by treatment interactions were found, the authors discuss the possibilities of an intervention that fosters a resource-oriented mindset focusing on solutions rather than problems. Moreover, Tang et al. examine the linkages between family factors at the whole, dyadic, and individual levels and two dimensions (affective and behavioral) of Oppositional Defiant Disorder symptoms among Chinese children. In their analyses, the most proximal factors (parent-child relationship and child emotion regulation, which were directly related to subsequent child internalizing problems) were significantly related to child behavioral defiant symptoms.

Finally, in this special topic there is also room for methodological discussions. Trying to integrate two very different worlds, Ray et al. explore the possibilities of functional neuroimaging at interpersonal and group levels. These authors attempt to embed their findings within classic concepts such as ‘collective consciousness’ or ‘crowd’. Furthermore, Buckley et al. report results from a post-programme survey of participants in a non-profit outdoor health programme predominantly targeted at women with families. In this study they test their hypothesis that the population-scale distribution of interest in nature exposure may be bimodal rather than unimodal. Under the bimodal view some individuals would be heavily addicted to nature-based outdoor activities, and others indifferent or indeed repelled by them.

## Author contributions

The author confirms being the sole contributor of this work and approved it for publication.

### Conflict of interest statement

The author declares that the research was conducted in the absence of any commercial or financial relationships that could be construed as a potential conflict of interest.
